# Blood markers of fibrinolysis and endothelial activation in canine babesiosis

**DOI:** 10.1186/s12917-017-0995-6

**Published:** 2017-03-31

**Authors:** Josipa Kuleš, Jelena Gotić, Vladimir Mrljak, Renata Barić Rafaj

**Affiliations:** 1 ERA Chair team VetMedZg, Internal Diseases Clinic, Faculty of Veterinary Medicine, University of Zagreb, Heinzelova 55, 10 000 Zagreb, Croatia; 2 Internal Diseases Clinic, Faculty of Veterinary Medicine, University of Zagreb, Heinzelova 55, 10 000 Zagreb, Croatia; 3 Department of Chemistry and Biochemistry, Faculty of Veterinary Medicine, University of Zagreb, Heinzelova 55, 10 000 Zagreb, Croatia

**Keywords:** Biomarkers, Hemostasis, Vascular Endothelium

## Abstract

**Background:**

Canine babesiosis is a tick-borne disease caused by hemoprotozoan parasites of the genus *Babesia.* The disease can be clinically classified into uncomplicated and complicated forms. The aim of this study was to assess the level of endothelial activation and alterations in the fibrinolytic pathway during canine babesiosis.

**Results:**

Blood samples were collected on the day of admission and on the 6th day after treatment with imidocarb propionate, from 30 dogs of various breeds and of both sexes with naturally occurring babesiosis caused by *B. canis.* In this prospective study, plasminogen activity was assessed using a chromogenic assay, and concentrations of high mobility group box-1 protein (HMGB-1), intercellular adhesive molecule-1 (ICAM-1), vascular adhesive molecule-1 (VCAM-1), soluble urokinase receptor of plasminogen activator (suPAR), thrombin activatable fibrinolysis inhibitor (TAFI), soluble thrombomodulin (TM) and plasminogen activator inhibitor-1 (PAI-1) were determined using a canine specific ELISA. Concentrations of TM, HMGB-1, VCAM-1 and suPAR were increased in dogs with babesiosis at admission compared to healthy dogs. After treatment, concentrations of TM were lower in infected dogs compared to healthy dogs. Dogs with babesiosis also had increased concentrations of TM, ICAM-1 and HMGB-1 and decreased plasminogen and PAI-1 at presentation compared to day 6 after treatment. Dogs with complicated babesiosis had higher concentrations of TM, HMGB1 and TAFI at admission compared to the 6th day.

**Conclusions:**

Biomarkers of endothelial activation and fibrinolysis were altered in dogs with babesiosis. Further studies into their usefulness as biomarkers of disease severity or prognosis is warranted.

## Background

Canine babesiosis is a tick-borne disease caused by hemoprotozoan parasites of the genus *Babesia* [1]. Three main species of large *Babesia* infect dogs, namely *B. vogeli*, *B. canis* and *B. rossi* [2]*.* The disease can be clinically classified into uncomplicated and complicated forms with a wide range of clinical presentations from a mild, subclinical illness to complicated forms and death [3].

Inflammation and hemostasis are tightly interrelated pathophysiologic processes that may affect each other considerably. Extensive crosstalk between immune and hemostatic systems occurs at the level of all components of the hemostatic system, including vascular endothelial cells, platelets, plasma coagulation cascade, physiologic anticoagulant pathways and fibrinolytic activity [4]. The presence of hemostatic abnormalities [5–8] and alterations in the inflammatory response [9–11] are well documented in canine babesiosis. However, whether there are alterations in the fibrinolytic system has not been previously examined.

Endothelial cell activation can be damaging if it is uncontrolled, persistent or widespread [12]. Endothelial dysfunction contributes to the pathogenesis of a variety of potentially serious infectious diseases and syndromes [13]. The vascular endothelium has an important role in the pathogenesis of canine babesiosis [6]. Differential cell surface molecule expression between quiescent and activated endothelial cells influences the degree of adhesion of circulating blood cells, as well as the relative balance between pro- and anti-coagulant activity [14]. Given their specificity for endothelial cells in the activated state, the soluble forms of cell-surface molecules, shed from endothelial cells after activation, have been widely studied as diagnostic and prognostic markers in a variety of infectious diseases. Intercellular adhesive molecule-1 (ICAM-1) mediates the firm adhesion of leukocytes to the endothelium and the subsequent transmigration to inflammatory sites, while vascular adhesive molecule-1 (VCAM-1) contributes to the adhesion of activated lymphocytes and monocytes to endothelial cells in acute inflammatory tissues [15]. Thrombomodulin (TM) is a an endothelial receptor that prevents dissemination of pro-coagulant and proinflammatory molecules, and by doing so, allows these molecules to act locally at the site of injury [16]. TM promotes thrombin-mediated activation of protein C and is essential for thrombin activatable fibrinolysis inhibitor (TAFI) activation. High mobility group box-1 protein (HMGB-1) is a highly conserved component of eukaryotic nuclei and is known as a DNA binding protein [17]. HMGB-1 is actively secreted by certain inflammatory cells and endothelial cells, and is passively released by necrotic or damaged cells [18]. Once released into the intravascular space, HMGB1 amplifies local inflammatory responses by enhancing the release of cytokines and chemokines from monocytes-macrophages and interacts with endothelial cells by up-regulating surface receptors and inducing the secretion of soluble proinflammatory mediators [19].

Components of the fibrinolytic system are mainly synthesized in the vascular endothelium and disturbances in vascular endothelium may induce an imbalance in fibrinolysis. Plasmin, as a result of plasminogen activation by plasminogen activators, cleaves fibrin and other important components of blood clots by initiating a proteolytic cascade leading to fibrinolysis and thrombolysis [20]. Tissue plasminogen activator (tPA) and urokinase plasminogen activator (uPA) are the most important mediators of plasminogen activation. Inhibition of the fibrinolytic system may occur at the level of plasminogen activation, mainly by a specific plasminogen activator inhibitor 1 (PAI-1) or by thrombin-activatable fibrinolysis inhibitor (TAFI), and at the level of plasmin, mainly by α2-antiplasmin [21]. It has been shown that bacteria, viruses and parasites utilise the plasminogen activation system for their biological needs [22–25].

It has been reported that a proinflammatory state occurs in babesiosis that is associated with increased concentrations of markers of endothelial cell activation and altered hemostasis [6, 26]. Because canine babesiosis could be accompanied by endothelial dysfunction, the relationship between endothelial function and fibrinolytic balance needs to be examined in more detail. Biomarkers of activated endothelium might indicate increased endothelial dysfunction during canine babesiosis. Our overall hypothesis is that markers of endothelial cell activation and alterations in fibrinolysis are increased in dogs with babesiosis and that these markers may be useful in monitoring disease progression. The specific aims of this study were: i) to determine whether markers of endothelial cell activation and fibrinolytic activity are different in dogs infected with *B. canis* at the time of diagnosis (day 0) and 6 days after imidocarb treatment, compared to healthy controls; ii) whether there are differences on day 0 compared with day 6 among infected dogs; iii) and whether there are differences between dogs with complicated and uncomplicated infection.

## Methods

### Animals

Blood samples were collected from 30 dogs of various breeds and of both sexes with naturally occurring babesiosis caused by *B. canis*, who were admitted to the Internal Diseases Clinic, Faculty of Veterinary Medicine, University of Zagreb, Croatia. This study was approved by the Committee on the Ethics of the University of Zagreb, Faculty of Veterinary Medicine. There were 12 females and 18 males, aged from 2 months to 10 years (median, interquartile range: 24 months, 11–60 months). Half of all dogs were mixed breed, and additionally there were 4 Labrador Retrievers, 2 Golden Retrievers, 2 American Staffordshire Terriers, and one Croatian Sheepdog, Alpine Dachsbracke, German Shepard, Doberman, Siberian Husky, Irish Red Setter and Alaskan Malamute. The diagnosis of babesiosis was confirmed by demonstration of the parasites within the infected erythrocytes in thin blood smears stained with May-Grünwald-Giemsa stain. Species were confirmed using PCR, as described previously [8]. One dose (6 mg/kg) of imidocarb dipropionate (Imizol® 12%, Schering-Plough, Kenilworth, NJ, USA) was administered to all the dogs subcutaneously on the day of admission. As needed, other supportive therapy was given mostly to dogs with complicated babesiosis. Supportive therapy included 0.9% saline solution and metoclopramide to treat nausea and vomiting. No reference was found about possible effects of this supportive therapy on endothelium function or fibrinolysis, except changes in hematocrit as consenquence of fluid compensation. Therefore we hypothesised that these differences in treatment among dogs didn’t have significant effect on investigated parameters. Blood was collected on the day of admission (B0), and on the 6th day (B6) of treatment.

The control group consisted of 10 healthy dogs. At the time of enrollment, none of the healthy dogs had histories of previous illness. Routine hematologic and biochemical analysis with urianalysis were performed, and all of the obtained results were within reference ranges. PCR was performed as for infected dogs, to rule out sublinical infection. All dogs were mixed breed, aged from 2 to 10 years, 6 of them were males and 4 females.

Serum samples from dogs with babesiosis and healthy dogs were screened for simultaneous qualitative detection of circulating *D. immitis* antigen and antibodies, both immunoglobulin G and M, to *E. canis*, *B. burgdorferi* sensu lato and *A. phagocytophilum* with the SNAP®4Dx® test (IDEXX Laboratories, Westbrook, Maine, USA) and antibodies to *L. infantum* with the SNAP Leishmania test (IDEXX Laboratories). Dogs with evidence of coinfection were excluded from the study.

On the basis of clinical manifestations and laboratory data (hematologic and biochemical analysis with urianalysis) at admission, the affected dogs were divided into two groups, complicated (*N* = 11) and uncomplicated (*N* = 19) babesiosis. An animal was classified as complicated if one of the following criteria were fulfilled [27]: renal dysfunction (serum creatinine concentration of more than 155 μmol/L in the absence of pre-renal azotemia), hepatic involvement (both alanine aminotransferase (ALT) greater than 176 U/L and alkaline phosphatase (AP) greater than 360 U/L), central nervous system dysfunction (a score on the modified Glasgow coma scale of less than 9), respiratory system dysfunction (radiographic evidence of pulmonary oedema, or increased effort to breathe), pancreatic dysfunction (all samples with increased amylase and lipase values in our lab are routinely checked by IDEXX SNAP cPL test for canine pancreas-specific lipase) and muscular involvement (creatine phosphokinase (CPK) more than 600 U/L). The noted complications were muscular involvement (7/11 dogs), respiratory system dysfunction (4/11), pancreatic dysfunction (1/11) and hepatic dysfunction (1/11).

### Sample analysis

Blood was collected by jugular venipuncture, using the Vacutainer blood collection system (Becton, Dickinson and Co., Rutherford, NJ). Blood samples were drawn atraumatically into EDTA and trisodium citrate tubes. EDTA plasma was separated by centrifugation at 1000 × g at 4 °C for 15 min, within 1 h of collection, and citrated plasma by centrifugation at 2000 × g at 4 °C for 15 min. Aliquots of plasma were stored at −80 °C for 2 to 8 months before analysis. Previous studies showed stability of fibrinolytic variables, soluble cell adhesion molecules and endothelial markers after long-time storage at −80 °C [28–30]. Concentrations of HMGB-1, ICAM-1, VCAM-1, soluble urokinase receptor of plasminogen activator (suPAR) and TAFI were measured in EDTA plasma, while concentrations of TM, PAI-1 and plasminogen (PLG) activity were measured in citrate plasma.

Plasminogen activity was assessed using a chromogenic substrate test on the ACL 7000 analyzer (Instrumentation Laboratory, Milan, Italy) using reagents from that manufacturer. The assay was calibrated using a pool from 10 clinically healthy dogs. Canine-specific ELISA kits were used for following analytes: TM, ICAM-1, and HMGB-1 (USCN Life Science, Wuhan, China), PAI-1 (Blue Gene Biotech, Shanghai, China), and VCAM-1, TAFI and suPAR (Biotang Source International, Camarillo, USA). For ELISA analyses, intra-assays precision were CV < 10%, and inter-assays CV < 12%, while spike-recovery were 92–107%, according to the manufacturers.

### ICAM-1 correction

For any given quantity (ng) of soluble ICAM-1 in the circulating blood, anemic animals will distribute that amount over a larger volume of plasma compared with non-anemic animals, resulting in a lower measured plasma concentration. To correct for anemia the following formula was applied for all samples to convert the measured plasma concentration of ICAM-1 to the concentration expected in whole blood [31]:

$$ \mathrm{Blood}\kern0.37em \mathrm{ICAM}{\textstyle \hbox{-} }1\kern0.37em \left(\mathrm{ng}/\mathrm{mL}\kern0.37em \mathrm{blood}\right)=\kern0.37em \left(\mathrm{plasma}\kern0.37em \mathrm{ICAM}{\textstyle \hbox{-} }1\kern0.37em \left(\mathrm{ng}/\mathrm{ml}\kern0.37em \mathrm{plasma}\right)\right)\times \left(1{\textstyle \hbox{-}}\mathrm{Hct}/\%\right), $$

where Hct stands for hematocrit.

### Statistical analysis

Statistical analysis was performed using the statistical computer application, Statistica 8 (StatSoft Inc., Tulsa, OK). Distribution of data was tested by Kolmogorov-Smirnov test. Differences between healthy and diseased dogs were assessed by *t*-test for normally distributed data, and Mann-Whitney *U*-test for nonparametric data. Wilcoxon matched pairs test and dependent *t*-test were used to access differences between dependent samples (dogs with babesiosis at admission and on the 6th day). Spearmans rank test was used to access correlations. Differences with a *P*-value <0.05 were considered statistically significant.

## Results

Markers for endothelial activation, such as TM, HMGB-1 and VCAM-1, were significantly increased in dogs with babesiosis at admission compared to healthy dogs (Table 1). Comparing day 0 and day 6, increased concentrations of TM, ICAM-1 and HMGB-1 were found on day 0. On day 6, concentrations of TM were decreased compared to healthy dogs. In case of fibrinolysis markers, suPAR was significantly increased in dogs with babesiosis at admission compared to healthy dogs, while PLG activity and PAI-1 concentration were decreased compared to day 6.

**Table 1:** Concentrations of blood markers of fibrinolysis and endothelial activation in canine babesiosis

Parameter (unit)	Group	*N*	Mean ± SD / Median (interquartile range)	*P* value^*^ B0-B6	*P* value^**^ B0 - C	*P* value^***^ B6 - C
TM (ng/ml)	B0	30	3.53 (2.65–4.25)	<0.001	0.001	0.009
	B6	30	1.47 (1.31–1.78)			
	Control	10	1.98 (1.62–2.41)			
ICAM-1 (ng/ml)	B0	30	6.75 (5.07–10.19)	0.007	0.866	0.636
	B6	28	6.42 (4.44–9.91)			
	Control	10	7.59 (4.6–18.7)			
HMGB-1 (ng/ml)	B0	30	47.88 (34.12–88.1)	<0.001	<0.001	0.524
	B6	28	17.59 (12.33–43.88)			
	Control	10	15.71 (13.27–26.0)			
PLG (%)	B0	30	85.5 (82.0–105.0)	0.011	0.053	0.906
	B6	27	103.0 (92.0–131.0)			
	Control	10	104.0 (96.0–111.0)			
PAI-1 (pg/ml)	B0	30	184.1 (92.67–405.96)	0.036	0.054	0.272
	B6	28	324.06 (176.59–608.05)			
	Control	10	413.05 (371.78–540.97)			
TAFI (ng/ml)	B0	30	461.15 ± 80.80	0.072	0.795	0.217
	B6	26	511.91 ± 140.65			
	Control	10	453.92 ± 55.93			
suPAR (pg/ml)	B0	30	4311.63 (3893.05–4840.3)	0.091	0.001	0.520
	B6	26	3725.3 (3277.2–4673.55)			
	Control	10	3613.35 (3052.65–3893.05)			
VCAM-1 (ng/ml)	B0	30	480.66 ± 59.21	0.841	0.026	0.056
	B6	29	478.44 ± 74.09			
	Control	10	416.73 ± 113.4			

Concentrations of soluble thrombomodulin (TM), soluble intercellular adhesive molecule-1 (ICAM-1), high mobility group box-1 protein (HMGB-1), plasminogen (PLG), plasminogen activator inhibitor-1 (PAI-1), thrombin activatable fibrinolysis inhibitor (TAFI), soluble urokinase receptor of plasminogen activator (suPAR) and soluble vascular adhesive molecule-1 (VCAM-1) in dogs with babesiosis at admission (B0), on the 6th day (B6) and healthy dogs (Control)

^*^ *P* value for differences between dependent samples on the day 0 and 6 (Wilcoxon matched pairs test and dependent *t*-test)

^**^ *P* value for differences between dogs with babesiosis day 0 compared to control (Mann-Whitney and *t*-test)

^***^ *P* value for differences between dogs with babesiosis day 6 compared to control (Mann-Whitney and *t*-test)

Markers for endothelial activation, such as TM and HMGB1, were significantly increased in dogs with complicated babesiosis at admission compared to day 6, while compared to dogs with uncomplicated babesiosis VCAM-1 was increased. Dogs with uncomplicated babesiosis on day 0 had higher TM, ICAM-1 and HMGB1 concentrations compared to day 6. In case of fibrinolysis markers, dogs with complicated babesiosis had higher concentrations of TAFI at admission compared to day 6, as well as compared to dogs with uncomplicated babesiosis, while dogs with uncomplicated babesiosis on day 0 had lower PAI-1 concentrations and plasminogen activity compared to day 6 (Tables 2 and 3).

**Table 2:** Concentrations of blood markers of endothelial activation in complicated (CB) and uncomplicated (UB) canine babesiosis

Parameter (unit)	Group	*N*	Median (interquartile range)	*P* value (UB 0 – CB 0)	*P* value (UB 0 – UB 6)	*P* value (CB 0 – CB 6)	*P* value (UB 6 – CB 6)
TM (ng/ml)	UB, day 0	19	3,77 (2,02–4,58)	0,933	<0,001	0,003	0,471
	CB, day 0	11	3,45 (3,13–4,09)				
	UB, day 6	19	1,55 (1,23–1,94)				
	CB, day 6	11	1,39 (1,31–1,55)				
ICAM-1 (ng/ml)	UB, day 0	19	6,96 (4,95–10,19)	0,832	0,002	0,575	0,174
	CB, day 0	11	6,46 (5,07–11,34)				
	UB, day 6	18	6,24 (3,76–8,09)				
	CB, day 6	10	6,69 (4,94–47,68)				
HMGB-1 (ng/ml)	UB, day 0	19	44,93 (34,12–109,49)	0,899	<0,001	0,022	0,869
	CB, day 0	11	50,84 (29,31–88,1)				
	UB, day 6	18	19,89 (12,73–37,34)				
	CB, day 6	10	15,5 (10,52–73,25)				
VCAM-1 (ng/ml)	UB, day 0	19	451,25 (429,2–507,4)	0,004	0,199	0,093	0,247
	CB, day 0	11	489,6 (476,3–576,05)				
	UB, day 6	19	494,05 (432,15–547,6)				
	CB, day 6	10	471,88 (417,45–489,6)				

*TM* soluble thrombomodulin, *ICAM-1* soluble intercellular adhesive molecule-1, *HMGB-1* high mobility group box-1 protein, *VCAM-1* soluble vascular adhesive molecule-1

**Table 3:** Concentrations and activity of blood markers of fibrinolysis in complicated (CB) and uncomplicated (UB) canine babesiosis

Parameter (unit)	Group	*N*	Median (interquartile range)	*P* value (UB 0 – CB 0)	*P* value (UB 0 – UB 6)	*P* value (CB 0 – CB 6)	*P* value (UB 6 – CB 6)
PLG (%)	UB, day 0	19	85,0 (81,5–98,0)	0,517	0,019	0,169	0,789
	CB, day 0	11	92,5 (83,0–114,0)				
	UB, day 6	16	101,0 (91,5–125,5)				
	CB, day 6	11	104,0 (92,0–131,0)				
PAI-1 (pg/ml)	UB, day 0	19	255,49 (96,81–519,27)	0,445	0,031	0,594	0,059
	CB, day 0	11	148,19 (75,1–323,52)				
	UB, day 6	17	392,92 (321,44–700,34)				
	CB, day 6	11	230,09 (117,59–309,24)				
TAFI (ng/ml)	UB, day 0	19	481,1 (417,55–538,7)	0,232	0,877	0,037	0,020
	CB, day 0	11	440,7 (388,6–481,1)				
	UB, day 6	16	455,15 (397,3–518,58)				
	CB, day 6	10	561,6 (475,35–693,45)				
suPAR (pg/ml)	UB, day 0	19	4283,75 (3893,05–4729,15)	0,553	0,179	0,333	0,979
	CB, day 0	11	4450,9 (3893,05–5006,9)				
	UB, day 6	16	3725,3 (3305,23–4562,23)				
	CB, day 6	10	3697,33 (3164,95–5228,75)				

*PLG* plasminogen, *PAI-1* plasminogen activator inhibitor-1, *TAFI* thrombin activatable fibrinolysis inhibitor, *suPAR* soluble urokinase receptor of plasminogen activator

Correlations between parameters in dogs naturally infected with *Babesia canis* at admission and on day 6 are presented in Table 4. Significant correlations were found between HMGB-1 and ICAM-1, PAI-1 and ICAM-1 on day 0 and 6, between PAI-1 and TM on day 0, and between TAFI and TM on day 6.

**Table 4:** Correlation between parameters in dogs naturally infected with *Babesia canis canis* at admission (B0) and on the 6th day (B6) (Spearman rank order) (*P < 0.05)

B0	TM	ICAM-1	HMGB1	plg	PAI-1	TAFI	suPAR	VCAM-1
TM	1.000	−0.086	−0.168	−0.030	0.394*	−0.344	−0.180	0.328
ICAM-1		1.000	0.651*	0.199	0.430*	−0.038	−0.142	−0.016
HMGB1			1.000	0.071	0.330	0.148	−0.049	−0.223
plg				1.000	0.277	−0.105	0.095	−0.056
PAI-1					1.000	−0.229	−0.106	0.174
TAFI						1.000	0.336	0.067
suPAR							1.000	0.107
VCAM-1								1.000
B6	TM	ICAM-1	HMGB1	PLG	PAI-1	TAFI	suPAR	VCAM-1
TM	1.000	−0.188	−0.200	0.284	−0.161	−0.439*	0.275	−0.384
ICAM-1		1.000	0.711*	0.087	0.689*	0.252	−0.039	−0.143
HMGB1			1.000	−0.082	0.383	0.359	−0.228	−0.253
PLG				1.000	0.081	−0.027	−0.027	−0.131
PAI-1					1.000	−0.097	0.035	−0.131
TAFI						1.000	−0.153	0.130
suPAR							1.000	0.102
VCAM-1								1.000

## Discussion

In this study, markers of endothelial activation were found to be increased in dogs with babesiosis at admission compared to healthy dog controls (TM, HMGB-1 and VCAM-1) and compared to day 6 of treatment (TM, ICAM-1, and HMGB-1), as well as between dogs with complicated and uncomplicated babesiosis (TM, HMGB1, ICAM-1, and VCAM-1). Fibrinolysis markers were also altered in dogs with babesiosis at admission compared to healthy dog controls (suPAR), and compared to day 6 (PLG and PAI-1), as well as between dogs with complicated and uncomplicated babesiosis (PAI-1 and TAFI).

HMGB-1 has been shown to play a key role in the pathogenesis of inflammatory and autoimmune diseases in humans [32]. As a proinflammatory mediator, HMGB-1 induces expression of two key adhesion molecules, ICAM-1 and VCAM-1 [19]. A few reports are available on blood HMGB-1 concentrations in veterinary medicine, including dogs with systemic inflammatory response syndrome and dogs with lymphoma and canine prostate cancer [33–35]. In this study, concentrations of plasma HMGB-1 were increased in dogs with babesiosis compared to healthy dogs at admission, as well as on day 6 after treatment. Concentrations of VCAM-1 were increased in dogs with babesiosis at admission compared to healthy dogs, while ICAM-1 concentrations were increased in dogs with babesiosis at admission compared to day 6. An increase of ICAM-1 compared to healthy dogs was reported previously in canine babesiosis [6]. Dogs with complicated babesiosis at admission had higher concentrations of VCAM-1 compared to dogs with uncomplicated babesiosis. Expression levels of soluble adhesion molecules are also increased in animal models of sepsis [36, 37]. In malaria, increased margination and sequestration of neutrophils is explained by the increased expression of cell adhesion molecules (ICAM-1 and VCAM-1) [38]. This mechanism of neutropenia that has been postulated for malaria might also be applied to the neutropenia present in babesiosis [39]. An increase in circulating adhesion molecules might either result from increased cytokine-induced expression by endothelial cells, increased proteolytic cleavage of endothelial-bound adhesion molecules secondary to endothelial damage and/or reduced soluble molecule clearance [40]. In this study, a strong positive correlation between HMGB1 and ICAM-1 (*P* < 0.05, *r* = 0.711) was found. Thus, the study suggests that HMGB1 activates components necessary for recruitment, adhesion, and transmigration of leukocytes across an activated endothelium in babesiosis.

The fibrinolytic system plays a key role in maintenance of vascular potency and thrombolysis by dissolving fibrin, and it is also involved in several physiological and pathological processes, such as local inflammatory reactions, neoplastic invasion and tissue remodeling [20]. The current study found lower plasminogen activity in dogs with babesiosis at admission compared to activity on day 6 (*P* = 0.011). Various pathogens have plasminogen-binding capacity, playing a role in the pathogenicity of these agents. Plasminogen interaction with the surface of various parasites or with their secreted molecules has been shown [25]. Decreased plasminogen might therefore be the result of its increased use by the parasite and/or consumption due to the hypercoagulability reported in babesiosis [7]. The latter is strengthened by the fact that lower concentrations of fibrinolysis inhibitors, PAI-1 and TAFI (only in complicated babesiosis), were found in dogs with babesiosis at admission, followed by their increase on the 6th day, so it can be proposed that inhibition of fibrinolysis may be impaired in dogs with babesiosis.

Thrombin-activatable fibrinolysis inhibitor (TAFI) is the most recently discovered fibrinolysis inhibitor. TAFI is activated by thrombin, the key component of the coagulation system, either free or in complex with TM [41]. In dogs, TAFI activity has been described only in experimental models of thrombosis [42, 43] and in studies in dogs with various spontaneous diseases [44]. Higher TAFI concentrations were found in dogs with complicated babesiosis on day 6 compared to dogs with uncomplicated babesiosis and also compared to dogs with complicated babesiosis at admission. Low concentrations of TAFI at admission, together with low PLG concentration, may suggest consumption from increased fibrinolysis due to the hypercoagulable state present in babesiosis cases. By day 6 the TAFI concentration had returned to normal.

Concentrations of TM were increased in dogs with babesiosis at admission compared to day 6 and compared to healthy dogs, while on day 6, concentrations in dogs with babesiosis were decreased compared to healthy dogs. Increased concentration of soluble TM has been proposed as both a diagnostic and prognostic marker of endothelial activation [45]. One study found that plasma TM concentrations were significantly higher for dogs with leishmaniasis [46], especially those with severe clinical signs, suggesting that TM can be used as a non-invasive marker for endothelial activation in dogs. In malaria, TM concentrations are increased with infection and decline with convalescence [38]. After treatment, TM concentrations were also decreased in our study. It is known that inflammatory cytokines and elastase released from activated leukocytes reduce TM expression and cleave the molecule when activation of inflammatory processes takes place [47, 48], thus the same mechanism is also possible in canine babesiosis.

Plasminogen activator inhibitor-1 (PAI-1) is a primary physiological inhibitor of tPA and uPA [49]. Similarly to TAFI, concentrations of PAI-1 were lower in dogs with babesiosis at admission compared to day 6, with concentrations approaching normal values with time. These findings contribute to documentation of fibrinolysis inhibitors consumption and increased fibrinolytic activity in this study. Contrary to TAFI, changes in PAI-1 concentrations were more profound in uncomplicated cases of babesiosis, probably due to faster resolution of endothelial and hemostatic alterations than in complicated babesiosis. The increase of PAI-1 during the course of babesiosis could lead to suppression of fibrinolysis. This study found a positive correlation between PAI-1 and ICAM-1 (*P* < 0.05, *r* = 0.689) at both time points, demonstrating an association between markers of endothelial activation and fibrinolysis, showing that increased expression of ICAM-1 and PAI-1 might be a result of the proinflammatory state in babesiosis.

suPAR is a new and promising inflammatory biomarker for various infectious diseases in humans [50]. Dogs with babesiosis had a higher suPAR concentration at admission compared to healthy dogs. In veterinary medicine there are no studies on suPAR as a plasma marker. There are only two studies of uPAR expression in histological samples of the canine urinary tract and prostate, where they found increased uPAR expression in inflammatory and neoplastic tissue [51, 52]. Increased suPAR concentrations have been reported in people with viral, bacterial or parasitic infections, as well as with autoimmune diseases [53]. A few studies on malaria in people showed increased suPAR concentrations in plasma and increased uPAR expression on endothelial cells, suggesting that uPAR might be an additional adhesion molecule for parasitised erythrocytes [54, 55]. The acute phase response in babesiosis is triggered by the overproduction of inflammatory mediators and leads to activation of the coagulation cascade and endothelial activation [6], which might cause increased expression of uPAR. Increased expression of uPAR on endothelial cells may modulate vascular permeability as uPA binding and plasmin generation at the surface of endothelial cells induces loss of cell-cell contacts, retraction of endothelial cells and increased permeability [56], all of which might contribute to the pathogenesis of babesiosis. Finally, it has been demonstrated that hypoxia stimulates uPAR-expression on a protein and mRNA level in cultured endothelial cells [57], suggesting that sequestration-induced hypoxia, previously documented in babesiosis [58, 59], could also contribute to high circulating suPAR concentrations in babesiosis. Therefore, increased suPAR concentrations in babesiosis may be a reflection of an inflammatory response.

## Conclusions

Biomarkers of endothelial activation and fibrinolysis were altered in dogs with babesiosis. Markers of endothelial activation are increased in babesiosis as a reflection of host inflammatory response and shift the hemostatic activity towards the procoagulant state. Decreased plasminogen activity and lower concentrations of both fibrinolysis inhibitors at admission (PAI-1 and TAFI in complicated cases) might result from increased consumption and lead to increased fibrinolytic activity due to the procoagulant state in babesiosis. These biomarkers might be clinically useful as biomarkers of disease monitoring in babesiosis. Further studies into their usefulness as biomarkers of disease severity or prognosis are warranted. Furthermore, studies of the complex mechanisms linking inflammation, endothelial dysfunction and hemostatic systems deserve more attention in veterinary medicine.

## Abbreviations

ALT: Alanine aminotransferase
AP: Alkaline phosphatase
CPK: Creatine phosphokinase
Hct: Hematocrit
HMGB-1: High mobility group box-1 protein
ICAM-1: Soluble intercellular adhesive molecule-1
PAI-1: Plasminogen activator inhibitor-1
PLG: Plasminogen
suPAR: Soluble urokinase receptor of plasminogen activator
TAFI: Thrombin activatable fibrinolysis inhibitor
TM: Thrombomodulin
tPA: Tissue plasminogen activator
uPA: Urokinase plasminogen activator
VCAM-1: Soluble vascular adhesive molecule-1
